# Study of the Role of Lipoprotein Maturation Enzymes in the Pathogenesis of the Infection Caused by the *Streptococcus suis* Serotype 2 Sequence Type 25 North American Prototype Strain

**DOI:** 10.3390/pathogens12111325

**Published:** 2023-11-07

**Authors:** Servane Payen, David Roy, Masatoshi Okura, Mariela Segura, Marcelo Gottschalk

**Affiliations:** 1 Groupe de Recherche sur les Maladies Infectieuses en Production Animale (GREMIP) and Swine and Poultry Infectious Diseases Research Center (CRIPA), Department of Pathology and Microbiology, Faculty of Veterinary Medicine, University of Montreal, Saint-Hyacinthe, QC J2S 2M2, Canada; servane.payen@umontreal.ca (S.P.); mariela.segura@umontreal.ca (M.S.); 2 Centre de Recherche du Centre Hospitalier Universitaire de Sherbrooke, Sherbrooke, QC J1H 5N4, Canada; dav.roy@gmail.com; 3 Division of Transboundary Animal Disease Research, National Institute of Animal Health, National Agriculture and Food Research Organization, Kagoshima 891-0105, Japan; mokura@affrc.go.jp

**Keywords:** *Streptococcus suis*, serotype 2, North America, lipoprotein maturation enzymes, inflammation

## Abstract

*Streptococcus suis* serotype 2 is an important swine bacterial pathogen causing sudden death, septic shock, and meningitis. However, serotype 2 strains are phenotypically and genotypically heterogeneous and composed of a multitude of sequence types (STs) whose distributions greatly vary worldwide. It has been previously shown that the lipoprotein (LPP) maturation enzymes diacylglyceryl transferase (Lgt) and signal peptidase (Lsp) significantly modulate the inflammatory host response and play a differential role in virulence depending on the genetic background of the strain. Differently from Eurasian ST1/ST7 strains, the capsular polysaccharide of a North American *S. suis* serotype 2 ST25 representative strain only partially masks sub-capsular domains and bacterial wall components. Thus, our hypothesis is that since LPPs would be more surface exposed in ST25 strains than in their ST1 or ST7 counterparts, the maturation enzymes would play a more important role in the pathogenesis of the infection caused by the North American strain. Using isogenic Δ*lgt* and Δ*lsp* mutants derived from the wild-type ST25 strain, our studies suggest that these enzymes do not seem to play a role in the interaction between *S. suis* and epithelial and endothelial cells, regardless of the genetics background of the strain used. However, a role in the formation of biofilms (also independently of the STs) has been demonstrated. Moreover, the involvement of LPP dendritic cell activation in vitro seems to be somehow more pronounced with the ST25 strain. Finally, the Lgt enzyme seems to play a more important role in the virulence of the ST25 strain. Although some differences between STs could be observed, our original hypothesis that LPPs would be significantly more important in ST25 strains due to a better bacterial surface exposition could not be confirmed.

## 1. Introduction

*Streptococcus suis* is one of the most important bacterial swine pathogens. It has been associated with a variety of infections including meningitis, septicemia, arthritis, and endocarditis. *S. suis* is also an emerging zoonotic agent causing mainly meningitis and septicemia with or without septic shock [1]. A total of 29 serotypes have been described and are defined based on the antigenicity of their capsular polysaccharide (CPS). Among them, serotype 2 is the most frequent type isolated from diseased pigs and humans worldwide [2,3]. However, evidence accumulated throughout the years has demonstrated a high level of genetic and phenotypic diversity within the *S. suis* species and serotypes. Therefore, serotype 2 strains are heterogeneous and belong to numerous sequence types (STs), as determined using multilocus sequence typing with the highly virulent ST1 predominating in Eurasia, the epidemic virulent ST7 in China, and the virulent ST25 and lower virulent ST28 in Canada and the USA [2,3,4,5]. Albeit these important differences, studies on the pathogenesis of the infection have dominantly used Eurasian strains, even though ST25 strains account for nearly 50% of serotype 2 isolates recovered from diseased pigs in Canada [6].

Although available information on the pathogenesis of the infection caused by *S. suis* has improved in recent years, knowledge about the mechanisms by which *S. suis* induces disease remains incomplete [1]. A variety of virulence factors have been proposed to be involved in the *S. suis* pathogenesis, including the CPS, cell-wall proteins, the suilysin, lipoteichoic acid modifications, and lipoproteins (LPPs) [7]. The majority of these factors have been described using Eurasian ST1 or ST7 serotype 2 strains. LPPs, a major class of surface proteins of this and other bacterial pathogens [8], are mainly anchored in the outer leaflet of the cytoplasmic membrane. Bacterial LPPs have been shown to perform various roles, such as nutrient uptake, signal transduction as well as a participation in antibiotic resistance and transport systems (such as ABC transporters) [9]. In addition, they have been shown to play a direct role in virulence-associated functions, such as colonization, adhesion and invasion, evasion of host defense, and immunomodulation [8,10,11,12,13]. LPPs are first translated as preprolipoproteins, which possess an N-terminal signal peptide with typical characteristic features of the signal peptides of secreted proteins. A conserved sequence at the C region of the signal peptides, referred to as lipobox, is modified through the covalent attachment of a diacylglycerol moiety to the thiol group of the cysteine residue. This modification is catalyzed by the enzyme lipoprotein diacylglyceryl transferase (Lgt), resulting in a prolipoprotein. After lipidation, lipoprotein signal peptidase (Lsp) is responsible for cleaving the signal sequence of the lipidated prolipoprotein and leaves the cysteine of the lipobox as the new amino-terminal residue, resulting in mature LPPs [14,15].

A recent study suggests that LPP maturation in *S. suis* regulates dendritic cell activation in vitro and host activation after infection. In addition, these enzymes seem to play a differential role in virulence depending on the genetic background of the strain (ST1 vs. ST7) [8]. It has been reported that, as different from Eurasian strains, the CPS of a North American *S. suis* serotype 2 ST25 representative strain only partially masks sub-capsular domains and bacterial wall components [16,17,18]. Thus, our hypothesis is that LPP maturation enzymes would be more surface exposed in ST25 strains than in their ST1 or ST7 counterparts, playing a more important role in the pathogenesis of the infection. In the present work, the role of the Lgt and Lsp enzymes in bacterial adhesion/invasion of porcine epithelial and endothelial cells, biofilm formation, in vitro and in vivo induction of inflammatory mediators, and virulence of the *S. suis* serotype 2 ST25 strain have been studied.

## 2. Materials and Methods

### 2.1. Bacterial Strains and Growth Conditions

The strains and plasmids used in this study are listed in Table 1. The wild-type *S. suis* serotype 2 strains P1/7 (ST1) and North American 89-1591 (ST25), previously used in our studies [8,17], and their lipoprotein maturation isogenic mutants previously obtained (P1/7) or those generated in the current study (89-1591) are also listed in Table 1. *S. suis* strains were cultured in Todd Hewitt broth (THB; Becton Dickinson, Mississauga, ON, Canada) as previously described [19]. The coagglutination test was performed with a standard technique [20]. Non-encapsulated CPS mutants (*cspF*) derived from strain P1/7 and 89-1591 were used as positive controls for some experiments [16]. *Escherichia coli* strains and different plasmids used in this study are also listed in Table 1. For in vitro cell culture assays, bacteria were prepared as previously described and resuspended in cell culture medium. When needed, antibiotics (Sigma-Aldrich, Oakville, ON, Canada) were added to the media at the following concentrations: for *S. suis*, spectinomycin (Spc) at 100 μg/mL; for *E. coli*, kanamycin (Km) and spectinomycin at 50 μg/mL and ampicillin (Ap) at 100 μg/mL.

### 2.2. DNA Manipulations

Genomic DNA was extracted from the *S. suis* wild-type strain 89-1591 using InstaGene Matrix solution (BioRad Laboratories, Hercules, CA, USA). Mini preparations of recombinant plasmids were carried out using the QIAprep Spin Miniprep Kit (Qiagen, Valencia, CA, USA). Restriction enzymes and DNA-modifying enzymes (Fisher Scientific, Ottawa, ON, Canada) were used according to the manufacturer’s recommendations. Oligonucleotide primers (Table 2) were obtained from Integrated DNA Technologies (Coralville, IA, USA) and PCRs carried out with iProof proofreading DNA polymerase (BioRad Laboratories, Mississauga, ON, Canada) or Taq DNA polymerase (Qiagen). Amplification products were purified using the QIAquick PCR Purification Kit (Qiagen) and sequenced using an ABI 310 Automated DNA Sequencer and ABI PRISM Dye Terminator Cycle Sequencing Kit (Applied Biosystems, Carlsbad, CA, USA).

### 2.3. Construction of the Lipoprotein Maturation Isogenic Mutants

Precise in-frame deletion of *lgt* and *lsp* genes from strain 89-1591 were constructed using splicing-by-overlap-extension PCRs as previously described [27,28]. Overlapping PCR products were cloned into pCR2.1 (Invitrogen, Burlington, ON, Canada), extracted with EcoRI, recloned into the thermosensitive *E. coli*–*S. suis* shuttle plasmid pSET4s, and digested with the same enzyme, giving rise to the knockout vector p4Δ*lgt* or p4Δ*lps*. Electroporation of the wild-type strain 89-1591 and procedures for isolation of the mutants were previously described [25]. Allelic replacement was confirmed by PCR and DNA sequencing analyses. Amplification products were purified with the QIAgen PCR Purification Kit (Qiagen) and sequenced as described above.

### 2.4. Complementation of the Mutants

The pMX1 vector was used for the generation of recombinant plasmids for complementation (Table 1). This vector is a derivative of the *E. coli*–*S. suis* shuttle cloning vector pSET2 [28] and possesses the *S. suis* malX promoter for transgene expression in *S. suis*. The entire *lgt* and *lsp* genes were amplified from genomic DNA of the *S. suis* 89-1591 strain and cloned into pMX1 via EcoRI and NcoI sites, generating complementation vectors pMX1-*lgt* and pMX1-*lsp*. These plasmids were introduced into *E. coli* MC1061 for verification of the sequences and then into the respective deletion mutants derived from *S. suis* 89-1591 to construct *lgt*- and *lsp*-complemented mutants.

### 2.5. Growth Analysis

Overnight cultures of wild-type and mutant strains were diluted in fresh THB or plasma and bacterial growth was followed during 24 h of incubation at 37 °C. The total number of CFU/mL was evaluated at different incubation times.

### 2.6. Bacterial Surface Hydrophobicity Assay

Relative surface hydrophobicity of the *S. suis* wild-type strain and its non-encapsulated mutant (positive control) was determined by measuring adsorption to *n*-hexadecane as previously described [16].

### 2.7. Bacterial Adhesion and Invasion Assays Using Porcine Brain Microvascular Endothelial and Tracheal Epithelial Cells

The porcine brain microvascular endothelial cell line (PBMEC; kindly provided by Dr. A. Friedl) [29] and the neonatal porcine tracheal epithelial cell line (NPTr; kindly provided by Dr. M. Jacques) [30,31] were used and cultured until confluence as previously described [16,32,33,34,35]. Cells were infected with 1 × 10^6^ CFU/well (multiplicity of infection or MOI = 10) of the different *S. suis* strains and incubated for 2 h at 37 °C in 5% CO_2_. The adhesion assay, which quantifies total cell-associated bacteria (surface-adherent and intracellular bacteria), and invasion assay (using the antibiotic protection assay) were performed as previously described [16,32,33,34,35].

### 2.8. Biofilm Assay

A biofilm assay was performed as previously described [36]. Briefly, strains were grown in basal broth medium that contained 0.5% glucose, 2% peptone (proteose peptone no. 3; BBL Microbiology Systems, Mississauga, ON, Canada), 0.3% K_2_HPO_4_, 0.2% KH_2_PO_4_, 0.01% MgSO_4_·7H_2_O, 0.002% MnSO_4_·6H_2_O, and 0.5% NaCl. Overnight cultures of *S. suis* were diluted in fresh culture broth to obtain an optical density at 655 nm (OD_655_) of 0.2. Samples (100 μL) were added to the wells of a Falcon^®^ 96-well polystyrene tissue culture plate (Corning, NY, USA) containing 100 µL of culture medium. Biofilm formation capacity was determined as previously described in the absence or presence of 5 mg/mL of porcine fibrinogen (Sigma-Aldrich). The plates were incubated for 24 h at 37 °C, and bacterial growth was evaluated by determining the OD_655_ using a microplate reader. Medium and free-floating bacteria were then removed, and biofilms were stained with crystal violet dye as previously described [36].

### 2.9. Generation of Bone-Marrow-Derived Dendritic Cells (bmDCs)

The femur and tibia from C57BL/6J mice (Jackson Research Laboratories, Bar Harbour, MA, USA) were used to generate bmDCs, as previously described [22]. Briefly, hematopoietic bone marrow stem cells were cultured in complete culture medium (RPMI-1640 supplemented with 5% heat-inactivated fetal bovine serum, 10 mM HEPES, 2 mM L-glutamine, and 50 µM 2-mercaptoethanol) (Gibco, Burlington, ON, Canada) and complemented with 20% granulocyte-macrophages colony-stimulating factor from mouse-transfected Ag8653 cells [37]. Cell purity was confirmed to be at least 90% CD11c+ by flow cytometry as previously described [22]. Albeit this culture system cannot completely rule out the presence of other innate cells such as macrophages, it represents an enriched source of bmDCs [17].

### 2.10. Preparation of Heat-Killed S. suis

Heat-killed *S. suis* suspensions were prepared as previously described [38]. Briefly, *S. suis* was cultured to the mid-log phase and then incubated at 60 °C for 45 min. Cultures were confirmed to be killed following culture on blood agar plates at 37 °C for 48 h. Heat-killed *S. suis* were re-suspended in cell culture medium at a concentration equivalent to 2 × 10^9^ CFU/mL prior to bone marrow dendritic cell stimulation. All manipulations were performed using endotoxin-free material (lipopolysaccharide).

### 2.11. Preparation of Bacterial Supernatant

Bacterial supernatants were prepared as previously described [8]. Briefly, bacteria were grown to the mid-log phase (OD_600nm_ = 0.6), and growth was immediately stopped on ice. Bacterial cultures were appropriately diluted and plated on THB agar (THA) to accurately determine bacterial concentrations. Then, bacteria were centrifuged for 15 min at 3312× *g* at 4 °C to separate bacteria from medium. Supernatants were collected and filtered using 0.2 µm filters. Supernatants were then applied to an Amicon^®^ Ultra-15 10K (Sigma-Aldrich) centrifugal filter and resuspended in cell culture medium prior to bone marrow dendritic cell stimulation. Absence of bacteria in supernatants was confirmed after culture on blood agar plates at 37 °C for 48 h. All manipulations were performed using endotoxin (lipopolysaccharide)-free material.

### 2.12. S. suis Activation of bmDCs

Cells were activated with either live bacteria, killed bacteria, or bacterial-free supernatant. All experiments were performed in the absence of endotoxin contamination and under non-toxic conditions, the latter being evaluated by the lactate dehydrogenase release with the CytoTox 96^®^ Non-Radioactive Cytotoxicity Assay (Promega, Madison, WI, USA) [19]. Prior to infection, cells were resuspended at 1 × 10^6^ cells/mL in complete medium and stimulated with the different live *S. suis* strains (1 × 10^6^ CFU/mL; initial MOI = 1). Conditions used were based on those previously published [8,19]. A higher MOI (corresponding to 100 = 1, before inactivation) was used with killed bacteria. Supernatants were collected at 4 h, 6 h, 8 h, 12 h, and 16 h following infection with *S. suis* (either live, heat-killed, or bacterial-free supernatant) [19]. Mock-infected cells served as negative controls. Secreted levels of tumor necrosis factor (TNF), interleukin (IL)-6, C-C motif chemokine ligand (CCL)3, and C-X-C motif chemokine ligand (CXCL)1 were quantified by sandwich ELISA using pair-matched antibodies from R&D Systems (Minneapolis, MN, USA) according to the manufacturer’s recommendations.

### 2.13. S. suis Virulence Mouse Model of Systemic Infection

A C57BL/6J mouse model of infection was used [39,40]. These studies were carried out in strict accordance with the recommendations of and approved by the University of Montreal Animal Welfare Committee guidelines and policies, including euthanasia to minimize animal suffering using humane endpoints, applied throughout this study when animals were seriously affected since mortality was not an endpoint measurement (permit number Rech-1570). Sixty 6-week-old female C57BL/6J (Jackson Research Laboratories) were used for these experiments (15 mice per group). Mice were inoculated with 5 × 10^7^ CFU via the intraperitoneal route and health and behavior monitored at least thrice daily until 72 h post-infection and twice thereafter until the end of the experiment (12 days post-infection) for the development of clinical signs of sepsis, such as depression, swollen eyes, rough hair coat, prostration, and lethargy. A second confirmatory trial with the wild-type strain and its Δ*lsp* mutant strain was repeated to confirm results, using 20 mice/group. For bacteremia studies, blood samples were collected from the caudal vein of surviving mice 12, 24, and 48 h post-infection and plated as previously described [40].

### 2.14. Measurement of Plasma (Systemic) Pro-Inflammatory Mediators

In parallel (a separate experiment), eight mice per group were intraperitoneally infected with 5 × 10^7^ CFU and blood was collected 12 h post-infection by intracardiac puncture following euthanasia and anti-coagulated with EDTA (Sigma-Aldrich) as previously described [39,40]. Plasma supernatants were collected following centrifugation at 10,000× *g* for 10 min at 4 °C and stored at −80 °C. The 12 h post-infection time point was selected to obtain maximal pro-inflammatory mediator production in the absence of significant mouse mortality as previously shown [40]. Plasmatic concentrations of IL-6, G-CSF, CCL2, CCL3, CCL4, CCL5, CXCL2, and CXCL9 were measured using a custom-made cytokine Bio-Plex Pro™ assay (Bio-Rad, Hercules, CA, USA) according to the manufacturer’s instructions. Acquisition was performed on the MAGPIX platform (Luminex^®^) and data were analyzed using the Bio-Plex Manager 6.1 software (Bio-Rad).

### 2.15. Statistical Analyses

The normality of data was verified using the Shapiro–Wilk test. Accordingly, parametric (unpaired *t*-test) or non-parametric tests (Mann–Whitney rank sum test), where appropriate, were performed to evaluate statistical differences between groups. The log-rank test was used to compare survival rates between wild-type-infected mice and those infected mutant strains. Each in vitro test was repeated in at least three independent experiments. A *p* < 0.05 was considered as statistically significant.

## 3. Results

### 3.1. Characteristics of the Δlgt and Δlsp Mutants Derived from the ST25 89-1591 Strain

**Figure 1:**
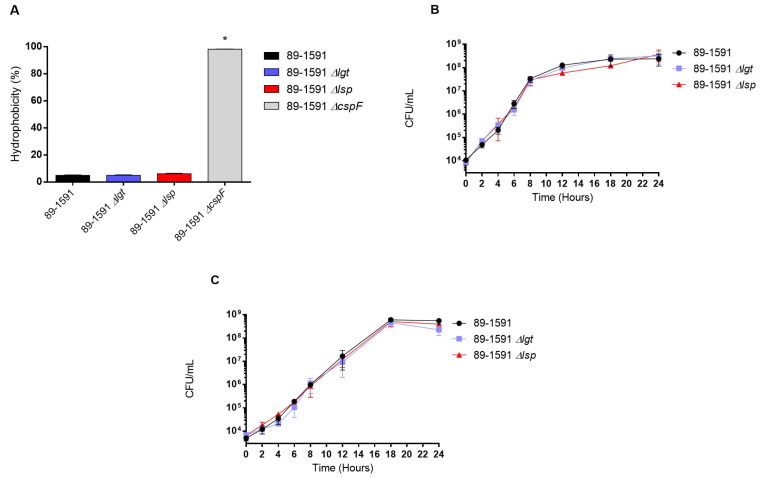
Bacterial hydrophobicity is not affected by the absence of Lgt or Lsp and both defective mutants grow as well as the wild-type strain in plasma. Surface hydrophobicity using *n*-hexadecane (**A**) and bacterial growth in THB (**B**) and plasma (**C**) of *S. suis* serotype 2 wild-type 89-1591 (ST25) strain, Δ*lgt* (blue), and Δ*lsp* (red) mutants (*n* = 3 independent repetitions for each experiment). The non-encapsulated 89-1591 Δ*cpsF* was used as a positive control for the hydrophobicity test. * (*p* < 0.05) indicates a significant difference between wild-type and Δ*cpsF* mutant strain.

The Δ*lgt* and Δ*lsp* mutants presented general phenotypic characteristics similar to ST25 wild-type strains. Both mutants remained well encapsulated as shown by the presence of hydrophobicity values between 4.9% and 6.1%, similar to the wild-type strain (4.9%) (Figure 1A). In addition, both mutants remained similarly serotypable (serotype 2) by the coagglutination test. Finally, the ST25 wild-type and Δ*lgt* and Δ*lsp* strains cultured in rich THB medium (Figure 1B) and in plasma (Figure 1C) presented similar good growth rates, as evaluated by bacterial counts.

### 3.2. Lack of Lipoprotein Maturation Enzymes Does Not Impair Adhesion to and Invasion of Respiratory Epithelial and Brain Microvascular Endothelial Swine Cells Regardless of the Sequence Type of the Strain

**Figure 2:**
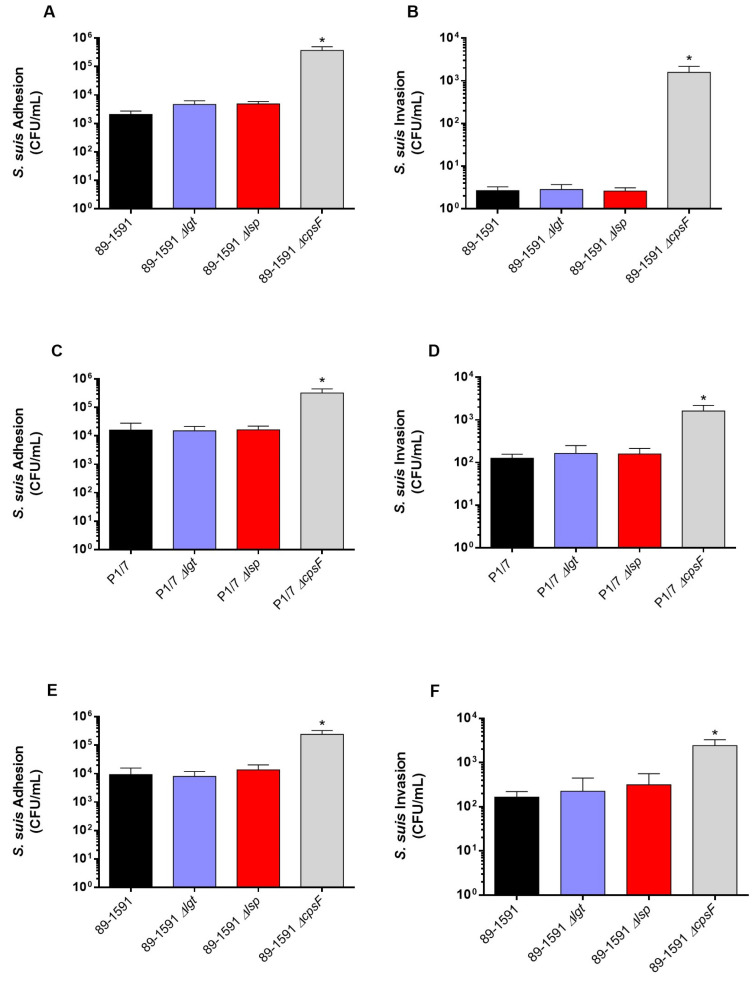
The lack of lipoprotein maturation enzymes does not change the capacity of adhesion and invasion of *S. suis* to swine epithelial and endothelial cells. Adhesion (**A**) and invasion (**B**) of the *S. suis* 2 wild-type strain 89-1591 (ST25) as well as its respective Δ*lgt* (blue) and Δ*lsp* (red) mutant strains to swine tracheal epithelial cells. Adhesion and invasion of the *S. suis* 2 wild-type P1/7 (ST1) strain (**C**,**D**) and 89-1591 (ST25) strain (**E**,**F**) as well as their respective Δ*lgt* (blue) and Δ*lsp* (red) mutant strains to brain microvascular endothelial cells. * Indicates a significant difference (*p* < 0.05). Each bar represents the mean bacterial concentration (CFU/mL) + SEM from at least three independent experiments.

We previously described that no differences were observed between the adhesion and invasion capacity of epithelial cells when ST1 and ST7 wild-type strains were compared to their respective lipoprotein maturation enzyme defective mutants [8]. However, since the cell-wall components of the ST25 strain are better surface exposed [16], LPPs may interplay differently with the host cells than ST1 and ST7 strains. Thus, the role of the Lgt and Lsp enzymes in the *S. suis* adhesion/invasion to NPTr and PBMEC was evaluated with the ST25 strain and its mutants. Interactions of the ST1 P1/7 strain (and the respective Δ*lgt* and Δ*lsp* mutants) with endothelial cells were also tested for the first time in the current study. For both cell types, and as expected, the non-encapsulated mutant strains (ST1 and ST25, positive controls) significantly adhered to and invaded cells more efficiently than their respective wild-type strains (Figure 2A–F). Concerning the epithelial cells, no differences were observed between the adhesion and invasion capacity of the ST25 wild-type strain and defective Lgt or Lsp mutants (Figure 2C,F). Similar results were obtained with PBMEC cells (Figure 2A,B,D,E). The ST1 P1/7 strain and its derived Δ*lgt* and Δ*lsp* mutants behaved similarly to the ST25 strains (Figure 2A–F). These results indicate that the Lgt and Lsp enzymes do not play a critical role in bacterial cell adhesion/invasion regardless of strain background and cell types tested.

### 3.3. Lgt and Lsp Enzymes Are Important for S. suis Biofilm Formation Regardless of the Sequence Type of the Strain

**Figure 3:**
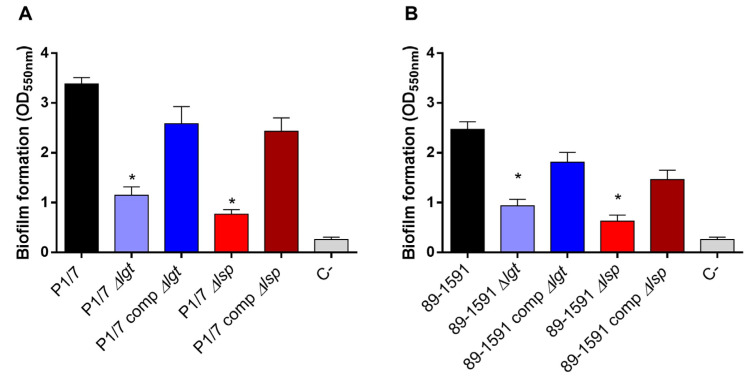
The Lgt and Lsp enzymes are both implicated in biofilm formation by *S. suis* independently of the ST of the strain. Biofilm formation capacity in the presence of porcine fibrinogen after 24 h of incubation at 37 °C of *S. suis* serotype 2 wild-type strain P1/7 (ST1) (**A**) and strain 89-1591 (ST25) (**B**) as well as their respective Δ*lgt* (blue), Δ*lsp* (red) mutant, the 89-1591 comp Δ*lgt* (dark blue), and 89-1591 comp Δ*lsp* complemented strains (dark red). Data represent the mean ± SEM from at least three independent experiments. * Indicates a significant difference with the respective wild-type strain (*p* < 0.05).

Biofilm formation by pathogenic microorganisms is a mechanism that allows them to become persistent colonizers and resist clearance by the innate and adaptive host immune system [36,41]. Since the involvement of LPPs in *S. suis* biofilm formation has been suggested [42], the ST1 and ST25 wild-type strains and their respective Δ*lgt* and Δ*lsp* mutant were tested using the crystal violet assay. Results confirmed that the wild-type ST1 strain produced more biofilm than the wild-type ST25 (Figure 3A,B) [43]. On the other hand, the Δ*lgt* and Δ*lsp* mutants of both strains showed a significantly lower capacity to form biofilm than their respective ST1 and ST25 wild-type strains (Figure 3A,B). The capacity to form biofilm was significantly restored in the complemented strains. Consequently, the LPP maturation enzymes play a role in the capacity to form biofilms regardless of the strain background.

### 3.4. The Diacyl Motif and the Peptide Signal Cleavage Are Important for the Recognition by Innate Immune Cells of Periplasmic and/or Secreted Lipoproteins of S. suis Serotype 2 ST25 Strain

**Figure 4:**
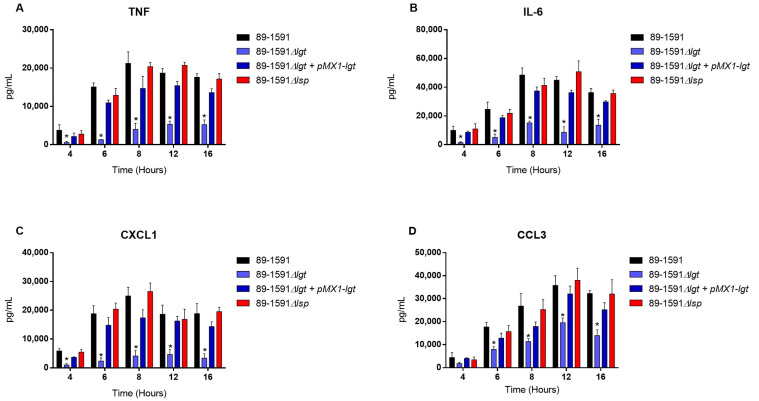
The diacyl motif is important for recognition by innate immune cells of periplasmic and/or secreted *S. suis* serotype 2 ST25 lipoproteins. Pro-inflammatory mediator production by bmDCs following activation with live bacteria of the *S. suis* serotype 2 wild-type strain 89-1591 (ST25) (**A**–**D**) (black), as well as their respective Δ*lgt* (blue) and Δ*lsp* (red)) mutant strains and the Δ*lgt* + pMX1-*lgt* complemented strain (dark blue). Production of TNF (**A**), IL-6 (**B**), CXCL1 (**C**), and CCL3 (**D**). Data represent the mean + SEM (*n* = 4 independent experiments). * *p* < 0.05 indicates a significant difference between the wild-type and mutant strains. Mock-infected cells induced negligible cytokine values < 300 pg/mL.

**Figure 5:**
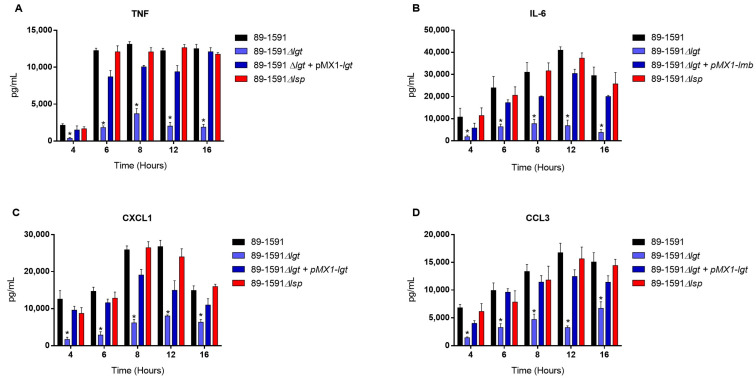
The diacyl motif is important for recognition by innate immune cells of periplasmic *S. suis* serotype 2 ST25 lipoproteins. Pro-inflammatory mediator production by bmDCs following infection with heat-killed bacteria of the *S. suis* serotype 2 wild-type ST25 strain 89-1591 (ST25) (**A**–**D**) (black), as well as its respective Δ*lgt* (blue) and Δ*lsp* (red)) mutant strains and the 89-1591 comp Δ*lgt* complemented strain (dark blue). Production of TNF (**A**), IL-6 (**B**), CXCL1 (**C**), and CCL3 (**D**). Data represent the mean + SEM (*n* = 4 independent experiments). * *p* < 0.05 indicates a significant difference between the wild-type and mutant strains. Mock-infected cells induced negligible cytokine values < 300 pg/mL.

**Figure 6:**
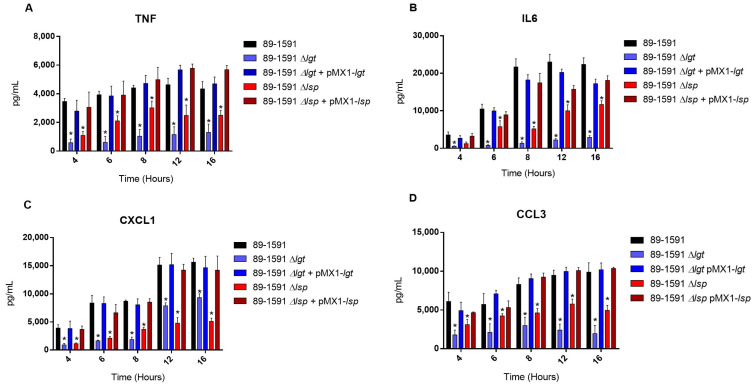
Both the diacyl motif and the peptide signal cleavage are important for recognition by innate immune cells of secreted *S. suis* serotype 2 ST25 lipoproteins. Pro-inflammatory mediator production by bmDCs following infection with bacterial-free supernatant of *S. suis* serotype wild-type ST25 strain 89-1591 (black) as well as its respective Δ*lgt* (blue) and Δ*lsp* (red) mutant strains and the 89-1591 comp Δ*lgt* (dark blue) or 89-1591 comp Δ*lsp* complemented strains (dark red). Production of TNF (**A**), IL-6 (**B**), CXCL1 (**C**), and CCL3 (**D**). Data represent the mean + SEM (*n* = 4 independent experiments). * *p* < 0.05 indicates a significant difference between wild-type and mutant strains. Mock-infected cells induced negligible cytokine values < 300 pg/mL.

The bmDC cells were used as an innate immune cell model given their critical role that they play during *S. suis* pathogenesis; in addition, their strong inflammatory response to *S. suis* activation has been well characterized [19,22]. In our previous study with ST1 and ST7 strains, we noticed the importance of the diacyl motif for the recognitions of membranes and secreted LPPs, and the peptide signal cleavage was shown to be similarly important for the recognition of secreted LPPs [8]. To investigate if the Lgt and Lsp enzymes of *S. suis* serotype 2 ST25 play a similar role, cells were activated for up to 16 h with either live bacteria (for evaluation of the role of periplasmic and possibly secreted LPPs) (Figure 4), heat-killed bacteria (for the evaluation of strict periplasmic LPPs) (Figure 5), or bacterial-free supernatant, to study the potential activation of strictly secreted LPPs (Figure 6).

For all experiments and all incubation times, control mock-infected cells presented negligible cytokine level values < 300 pg/mL. When using live bacteria, the ST25 Δ*lgt* mutant (but not the Δ*lsp* mutant) induced lower levels of the different pro-inflammatory mediators TNF, IL6, CXCL1, and CCL3 during, at least, 16 h of activation (Figure 4). Similar results were observed using washed heat-killed bacteria (Figure 5). Moreover, both the diacyl motif and the peptide signal cleavage were shown to be similarly important for the recognition of secreted LPPs up to 16 h of incubation (Figure 6). In all cases, complemented mutants restored the activation capacity of the strains, confirming the influence of the gene deletion (Figure 4, Figure 5 and Figure 6).

### 3.5. The Absence of Lgt Enzyme, but Not Lsp, Significantly Affects the Virulence of S. suis Serotype 2 ST25

**Figure 7:**
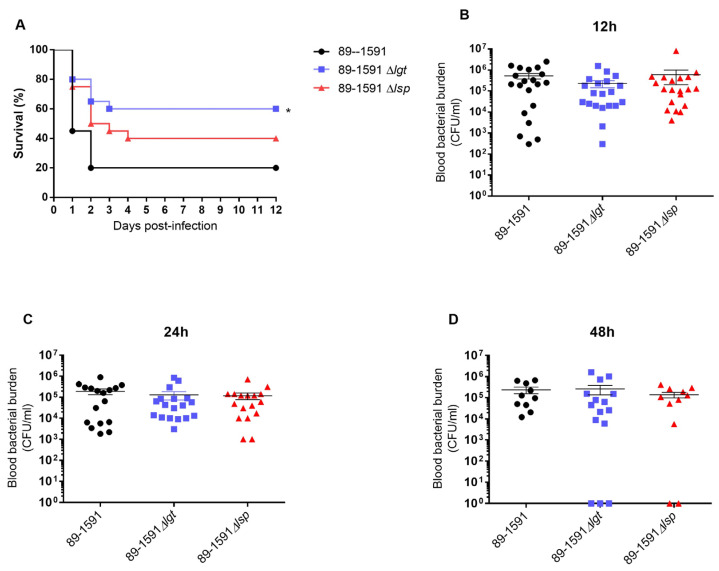
Presence of Lgt, but not Lsp, is significantly important for *S. suis* ST25 virulence but it does not affect bacteremia levels. Survival (**A**) and blood bacterial burden at 12, 24 h and 48 h post-infection (**B**–**D**) of C57BL/6 mice following intraperitoneal inoculation of the *S. suis* wild-type 89-1591 strain (ST25) (black), and Δ*lgt* (bleu) and Δ*lsp* (red) mutant strains. Data represent survival curves (**A**) (*n* = 15) or geometric mean (**B**–**D**) (*n* = survived mice at each time point). * *p* < 0.05 indicates a significant difference between survival of mice infected the wild-type and the Δ*lgt* mutant strain.

To confirm the role of Lgt and/or Lps in the ST25 strain on virulence and the development of clinical disease, a well-characterized C56BL/6 mouse model of infection was used. Mouse survival was better with mutant strains when compared to the wild-type strain. Surprisingly, only the absence of the Lgt, but not the Lsp, significantly affects the strain virulence (*p* < 0.05) (Figure 7A). Blood bacterial burden was evaluated at the early infection times of 12 h (Figure 7B), 24 h (Figure 7C), and 48 h (Figure 7D)post-infection and no differences were observed in the acute blood bacterial burden between mice infected for the wild-type or mutant strains.

### 3.6. Absence of the Lgt or Lsp Enzymes Significantly Reduces the In Vivo Inflammatory Response of Mice Infected with Either the Wild Type S. suis Serotype 2 Strain ST25 or Its Respective Δlgt or Δlsp Mutant

**Figure 8:**
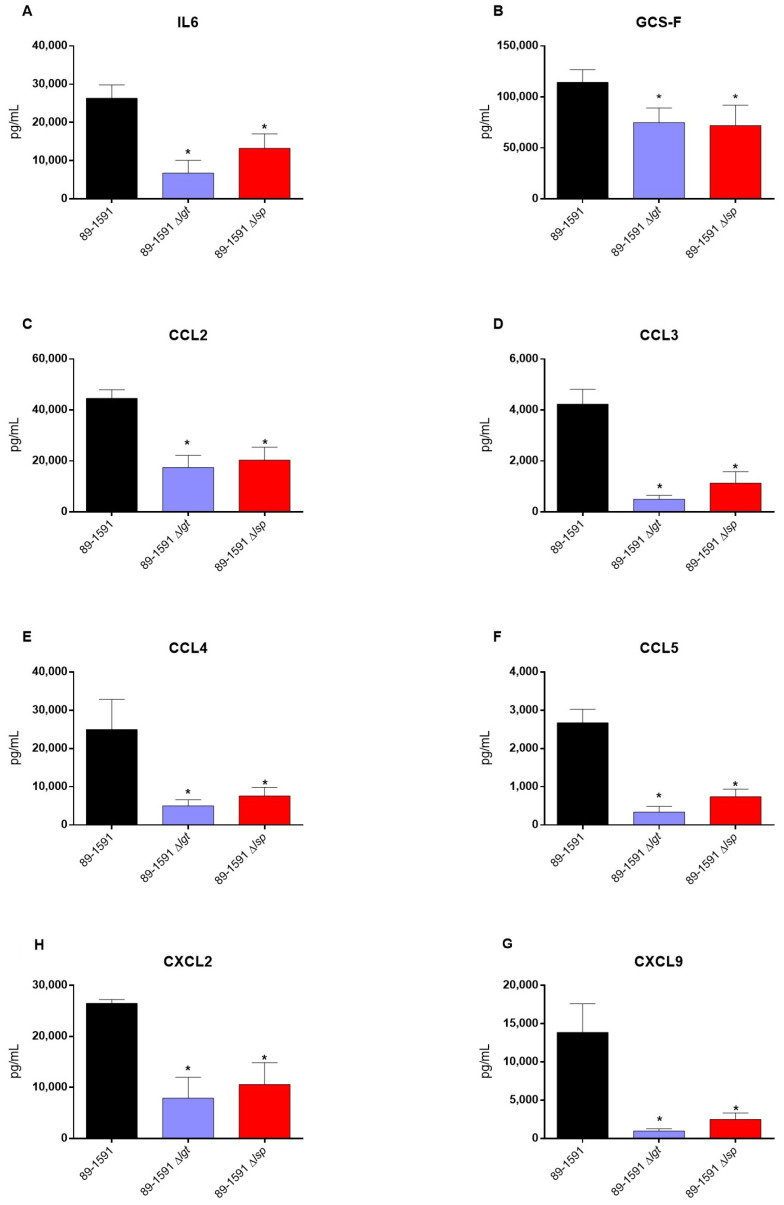
Lgt and Lsp enzymes are important for the recognition of lipoproteins and therefore for the establishment of the inflammatory response. Plasma levels of IL-6 (**A**), G-CSF (**B**), CCL2 (**C**), CCL3 (**D**), CCL4 (**E**), CCL5 (**F**), CXCL9 (**G**), and CXCL2 (**H**) in mice 12 h following intraperitoneal inoculation of *S. suis* serotype 2 wild-type ST25 strain 89-1591 (black) or its Δ*lgt* (blue) and Δ*lsp* (red) mutant strains. Data represent mean + SEM (*n* = 8 individuals). * *p* < 0.05 indicates a significant difference between plasma levels of mice infected with the mutant strains when compared to those infected with the wild-type strain.

The deletion of LPP maturation enzyme genes was previously described to be implicated in *S. suis* recognition by the immune system that may result in an exacerbated systemic inflammatory response in Eurasian ST1 and ST7 strains [8]. Consequently, for the effect of *lgt* and *lsp* gene deletion on the inflammatory response of animals infected with the ST25 wild-type and mutant strains, plasma mediators were evaluated after 12 h of infection (Figure 8). Concentrations of the all mediators tested (IL-6, IL-12p70, G-CSF, IFN-γ, CCL2, CCL3, CCL4, CCL5, CXCL2, and CXCL9) were significantly lower in mice infected with both mutants when compared to their respective ST25 wild-type strain (Figure 8).

## 4. Discussion

Evidence accumulated over the years demonstrated a high level of genetic diversity in the species *S. suis* [2,3]. However, most studies on the pathogenesis of the infection caused by *S. suis* serotype 2 have used Eurasian strains (virulent ST1 and/or ST7), which greatly differ from their North American counterparts, including ST25 strains. The pathogenesis of the infection seems to present a marked difference between ST25 and ST1/ST7 strains. For example, the suilysin, a virulence factor present in ST1/ST7 strains [44], is not produced by ST25 strains [45]. In addition, mechanisms of production of type I interferon and interleukin-1 during *S. suis* infection are also strain-dependent (ST1 vs. ST7 vs. ST25). Interaction of ST25 strains with phagocytic cells also differs from those of other STs [16]. Although LPP maturation enzymes possess a high level of amino acid homology, our recent studies showed that Lgt and Lsp play a differential role in virulence in Eurasian strains (ST1 and ST7) depending on the genetic background of the strain [8]. Since it has been reported that the CPS only partially masks sub-capsular domains and bacterial wall components of North American *S. suis* serotype 2 ST25 strains, we evaluated the role of LPP maturation enzymes in different aspects of the pathogenesis of the infection caused by the North American type [16].

Since several LPPs are known for being substrate-binding proteins of ABC transporter systems responsible for the acquisition of multiple nutrients [14], the maturation enzymes may play a role in bacterial growth under different conditions. For example, Lgt has been shown to be important for *Staphylococcus aureus* and *Streptococcus pneumoniae* growth in poor medium or in-vivo-like conditions [46,47]. Likewise, the growth of Lsp mutants was impaired in rich medium in *S. pneumoniae* and *Listeria monocytogenes* [48,49]. However, similarly to what has been described for ST1/ST7 strains, we showed that growth in both the rich medium and in-vivo-like conditions (plasma) of Δ*lgt* and Δ*lsp* in the ST25 mutant strain was not significantly affected. These results indicate that LPP maturation enzymes are not important for the growth of *S. suis* serotype 2 independently of the ST.

Results from our last study indicate that the adhesion and invasion capacity of *S. suis* to epithelial cells of ST1 and ST7 mutants defective in LPP maturation enzymes was not affected [8]; similar results have been obtained with the ST25 strain in the current study. Likewise, the absence of these enzymes does not affect *S. suis* adhesion/invasion to brain endothelial cells for the ST1 and ST25 strains. These results with different cells reinforce the concept that LPPs probably do not play important roles in the *S. suis* interactions with host cells. Another hypothesis includes the possibility that a lack of LPP maturation does not completely eliminate the functional activities of such proteins, as shown for *Streptococcus equi* [50]. Finally, since all our studies have been carried out with serotype 2 strains, a hypothetical role of the LPP maturation enzymes on bacterial–cell interactions in other *S. suis* serotypes cannot be ruled out. Previous studies showed that a single protein could play important or limited roles during the first steps of the pathogenesis of the infection depending on the serotype [51,52].

Alongside adhesion and invasion to host cells, the capacity to form biofilms has been described as being important for the pathogenesis of the infection caused by different pathogens, including *S. suis* [32,53]. Studies on the role of Lgt and/or Lsp on biofilm formation by streptococci and other pathogens are scarce. It has been reported that LPPs (such as VacJ) play an important role in biofilm formation by the swine pathogens *Glaesserella parasuis* and *Actinobacillus pleuropneumoniae* [54,55]. However, the role of LPPs in biofilm formation is not always clear. Although the LPP AdcA has been implicated in biofilm formation by *Streptococcus gordonii* [56], another study showed that Lgt negatively regulates biofilm formation for this bacterial species [57]. Similarly, in *E. coli*, the Rcs pathway through the outer membrane LPP RcsF reduces biofilm formation [58]. Our results showed that the absence of the Lgt and Lsp leads to a decrease in biofilm formation for both ST1 and ST25 strains, suggesting that LPPs are indeed involved in this process. Accordingly, AdcACB and Lmb, two zinc-binding LPPs, have already been described as implicated in *S. suis* biofilm formation [42]. However, the technique used in our studies only allows us to observe the overall behavior of the biofilm. Thus, it would be necessary to use other techniques that allow for a better understanding of the biofilm structuring process, and to shed the biofilm on a bacterial scale.

Studies previously reported that Lgt and Lsp in *S. suis* ST1 and ST7 strains are critical for the induction of an inflammatory response in vitro and in vivo, especially Lgt [8,59]. The diacylglycerol provided by the Lgt enzyme is the main motif recognized by the immune system in Gram-positive bacteria [60]. Both enzymes have been described as being critical for cell activation in other streptococci [12,47,50,61]. In the current study, we confirmed that lack of LPP maturation enzymes affects cell activation by the North American ST25 strain. Indeed, the role of such enzymes seems to be enhanced with this strain. In the previous study with ST1 and ST7 strains, levels of the different pro-inflammatory mediators were affected only at early incubation times when the mutant strains were tested [8]. The reduction in cytokine expression using the ST25 Δ*lgt* and/or Δ*lsp* mutants was clearly observed at least up to 16 h of incubation (maximum incubation time tested in the current study). Indeed, as mentioned, there are important phenotypic differences between the ST1/ST7 and ST25 strains, and putative virulence protein factors, such as the suilysin, the muramidase-released protein, and the extracellular protein factor, are present in the former strains only [52,62]. Even if their role in the pathogenesis of *S. suis* is not totally elucidated and they are not critical for the virulence, it is possible that these markers and other factors present in ST1/ST7 strains but absent in ST25 strains compensate for further cell activation [44]. Indeed, other surface or secreted proteins not regulated by the Lgt and Lsp enzymes are also cell activators [51,63]. It is important to mention that differences in cell activation by the mutant strains tested in closed in vitro systems do not always represent in vivo activation. In fact, both mutants induced significantly fewer pro-inflammatory cytokines than the wild-type strain after in vivo infection.

It has been previously described that the role of the LPP maturation enzymes on virulence was dependent on the strain background; indeed, the absence of the Lgt or Lsp enzymes reduced the virulence of an ST7 (but not that of an ST1) *S. suis* strain [8]. In the case of the *S. suis* serotype 2 ST25, only the Δ*lgt* mutant was significantly less virulent when compared to the wild-type strain, although the Δ*lsp* mutant presented a clear tendency to also be less virulent (*p* = 0.07). Reasons for the differences observed between the STs are not completely understood, although the hypothesis that the three strains may use different pathogenic mechanisms can be postulated [17,64]. The reduced virulence could be attributed, as mentioned above, to a lesser inflammatory reaction, since animals infected with any of the mutant strains induce significantly fewer pro-inflammatory cytokines. In addition, similar to what has been observed for ST1 and ST7 strains, the absence of the LPP maturation did not seem to affect bacterial survival in blood, indicating that the better mouse survival was not due to a reduced bacteremia. It can be concluded that LPP maturation is implicated in *S. suis* recognition by the immune system independently of the strain background. Finally, it should be considered that the infection used in the mouse model of systemic infection is five times higher with ST25 strain than that for ST1 and ST7 strains (due to a lower virulence potential). Although the final mortality rates obtained are similar, the comparison with other STs may be somehow biased.

## 5. Conclusions

Overall, our studies suggest that LPP maturation enzymes do not seem to play a role in the interaction between *S. suis* and epithelial and endothelial cells, regardless of the genetics background of the serotype 2 strain used. However, the potential involvement of LPPs in the formation of biofilms (also independently of the STs) has been demonstrated. Moreover, the involvement of such enzymes in dendritic cell activation in vitro seems to be more pronounced with the ST25 strain. Finally, the Lgt enzyme seems to play a more important role in the virulence of the ST25 strain. Although some differences between STs could be observed, our original hypothesis that LPP maturation enzymes would be more important in ST25 strains due to a better bacterial surface exposition could not be confirmed. However, since it has been reported that CPS composition is different depending on the serotype, LPP maturation enzymes could have a differential role in pathogenesis and virulence depending on the serotype; indeed, future studies are expected with other important serotypes such as serotypes 9 and 14.

## Figures and Tables

**Table 1:** List of strains and plasmids used in this study.

Strain or Plasmid	Characteristics	References
***Streptococcus suis***		
P1/7	Virulent serotype 2 ST1 strain isolated from a case of pig meningitis in the United Kingdom	[21]
P1/7 Δ*lgt*	Isogenic mutant derived from P1/7; in frame deletion of *lgt* gene	[8]
P1/7 Δ*lsp*	Isogenic mutant derived from P1/7; in frame deletion of *lsp* gene	[8]
P1/7 comp Δ*lgt*	Mutant Δ*lgt* complemented with pMX1-*lgt* complementation vector	[8]
P1/7 comp Δ*lsp*	Mutant Δ*lsp* complemented with pMX1-*lsp* complementation vector	[8]
P1/7 Δ*cpsF*	Isogenic mutant derived from P1/7; in frame deletion of *cpsF*	[22]
89-1591	Virulent North American ST25 strain isolated from a case of pig sepsis in Canada	[23]
89-1591 Δ*lgt*	Isogenic mutant derived from 89-1591; in frame deletion of *lgt* gene	This study
89-1591 Δ*lsp*	Isogenic mutant derived from 89-1591; in frame deletion of *lsp* gene	This study
89-1591 comp Δ*lgt*	Mutant Δ*lgt* complemented with pMX1-*lgt* complementation vector	This study
89-1591 comp Δ*lsp*	Mutant Δ*lsp* complemented with pMX1-*lsp* complementation vector	This study
89-1591 Δ*cpsF*	Isogenic mutant derived from 89-1591; in frame deletion of *cpsF*	[16]
***Escherichia coli***		
TOP10	F^−^ *mrcA* Δ(*mrr-hsd*RMS-*mcr*BC) φ80 *lacZ*ΔM15 Δ*lac*X74 *rec*A1 *ara*D139 Δ(*araleu*) 7697 *gal*U *gal*K *rps*L (Str^r^) *end*A1 *nup*G	Invitrogen
MC1061	F^−^ Δ(*ara-leu*)*7697* [*araD139*]B/r Δ(*codB*-*lacI*)3 *galK16 galE15* λ-e14-*mcrA0 relA1 rpsL150*(StrR) *spoT1 mcrB1 hsdR2*(r-m+)Host for pMX1 derivatives	[24]
**Plasmids**		
pCR2.1	Ap^r^, Km^r^, pUC *ori*, *lac*ZΔM15	Invitrogen
pSET4s	Spc^r^, pUC *ori*, thermosensitive pG+host3 *ori*, *lac*ZΔM15	[25]
pMX1	Replication functions of pSSU1, MCS pUC19 *lac*Z Spc^r^, *mal*X promoter of *S. suis*, derivative of pSET2	[25,26]
p4Δ*lgt*	pSET-4s carrying the construct for *lgt* allelic replacement	This study
p4Δ*lsp*	pSET-4s carrying the construct for *lsp* allelic replacement	This study
pMX1-*lgt* (P1/7)	pMX1 carrying intact *lgt* gene	[8]
pMX1-*lsp* (P1/7)	pMX1 carrying intact *lsp* gene	[8]
pMX1-*lgt* (89-1591)	pMX1 carrying intact *lgt* gene	This study
pMX1-*lsp* (89-1591)	pMX1 carrying intact *lsp* gene	This study

**Table 2:** List of oligonucleotide primers used in this study.

Name	Sequence (5′–3′)	Construct
*lgt*-ID1	GGAACGCTATGGAACAGGTC	p4Δ*lgt*
*lgt*-ID2	CACTCCATGAAAAGGCGACG	p4Δ*lgt*
*lgt*-ID3	CGTAGACGGCCAAAATTCC	p4Δ*lgt*
*lgt*-ID4	CGCTTATCTGCTGGATTCTCC	p4Δ*lgt*
*lgt*-ID5	GCCAATCGTCTGCATCAAGG	p4Δ*lgt*
*lgt*-ID6	GGGTTGATAGAATGGGATTGCATACCAACG	p4Δ*lgt*
*lgt*-ID7	CGTTGGTATGCAATCCCATTCTATCAACCC	p4Δ*lgt*
*lgt*-ID8	GACCGACTTGCTGGTCAAAC	p4Δ*lgt*
*lsp*-ID1	TGAGAAAACTGTTGTGGGTA	p4Δ*lsp*
*lsp*-ID2	AGAGCACCAGCAATCATCAA	p4Δ*lsp*
*lsp*-ID3	TTGATGATTGCTGGTGCTCT	p4Δ*lsp*
*lsp*-ID4	TAGACAGCGAACAGAGATAC	p4Δ*lsp*
*lsp*-ID5	TACGCTACGTTGTAGCCATTGC	p4Δ*lsp*
*lsp*-ID6	ACCTACACCAACTGTTAATACTACCATCAA	p4Δ*lsp*
*lsp*-ID7	TTGATGGTAGTATTAACAGTTGGTGTAGGT	p4Δ*lsp*
*lsp*-ID8	CGCGCTGCAGCCAAAGTGTAGTCACCAAAA	p4Δ*lsp*
pMX1-*lgt*-F	CCGCCATGGACAGATGGGGTTTGATGCAAC	pMX1-*lgt*
pMX1-*lgt*-R	CGCGAATTCGGACAAGGCAATAATCAAGAC	pMX1-*lgt*
pMX1-*lsp*-F	GTGCCATGGACTTTATTGAAACCATGCAGG	pMX1-*lsp*
pMX1-*lsp*-R	ATCGAATTCAATACCACCAACCTCAACTCT	pMX1-*lsp*

## Data Availability

The data presented in this study are available on request from the corresponding author.
